# Heparinoid Complex-Based Heparin-Binding Cytokines and Cell Delivery Carriers

**DOI:** 10.3390/molecules24244630

**Published:** 2019-12-17

**Authors:** Masayuki Ishihara, Shingo Nakamura, Yoko Sato, Tomohiro Takayama, Koichi Fukuda, Masanori Fujita, Kaoru Murakami, Hidetaka Yokoe

**Affiliations:** 1 Division of Biomedical Engineering, Research Institute, National Defense Medical College, 3-2 Namiki, Tokorazawa, Saitama 359-8513, Japan; snaka@ndmc.ac.jp (S.N.); ysato@ndmc.ac.jp (Y.S.); khf05707@nifty.com (K.F.); 2 Department of Oral and Maxillofacial Surgery, National Defense Medical College, 3-2 Namiki, Tokorozawa, Saitama 359-8513, Japan; taka01@ndmc.ac.jp (T.T.); murakami@ndmc.ac.jp (K.M.); yokoe@ndmc.ac.jp (H.Y.); 3 Division of Environmental Medicine, Research Institute, National Defense Medical College, 3-2 Namiki, Tokorozawa, Saitama 359-1324, Japan; fujitama@ndmc.ac.jp

**Keywords:** glycosaminoglycan, heparinoid, heparinoid-based biomaterials, heparin-binding cytokines, heparinoid-carrying polystyrene, polyelectrolyte complexes

## Abstract

Heparinoid is the generic term that is used for heparin, heparan sulfate (HS), and heparin-like molecules of animal or plant origin and synthetic derivatives of sulfated polysaccharides. Various biological activities of heparin/HS are attributed to their specific interaction and regulation with various heparin-binding cytokines, antithrombin (AT), and extracellular matrix (ECM) biomolecules. Specific domains with distinct saccharide sequences in heparin/HS mediate these interactions are mediated and require different highly sulfated saccharide sequences with different combinations of sulfated groups. Multivalent and cluster effects of the specific sulfated sequences in heparinoids are also important factors that control their interactions and biological activities. This review provides an overview of heparinoid-based biomaterials that offer novel means of engineering of various heparin-binding cytokine-delivery systems for biomedical applications and it focuses on our original studies on non-anticoagulant heparin-carrying polystyrene (NAC-HCPS) and polyelectrolyte complex-nano/microparticles (N/MPs), in addition to heparin-coating devices.

## 1. Introduction

Heparinoids are generically referred to as heparin, heparan sulfate (HS), and heparin-like molecules, and they are involved in various biological processes involving heparin-binding proteins, such as various cytokines. Heparinoids are a sub-group of glycosaminoglycans (GAGs) found in animal tissues. GAGs include other polysaccharides, such as hyaluronic acid (HA), chondroitin sulfate (CS), dermatan sulfate, and keratan sulfate, in addition to heparinoids, all of which bear negative charges that vary in density and position [1,2,3]. CS is formed by the repetitive unit of glucuronic acid linked β1→3 to a β-*N*-acetylgalactosamine. The galactosamine residues may be *O*-sulfated at the C-4 and/or C-6 position, but they contain no *N*-sulfated group [1,2,3]. These GAGs exhibit little anti-thrombotic activity, which is typically a specific feature of heparin. On the other hand, hexuronate residues in heparin/HS are present as either as β-d-glucuronate (GlcA) or the C-5 epimer, α-l-iduronate (IdoA). Heparin/HS basically consist of a disaccharide repeat of (1→4 linked) α-d-glucosamine (GlcN) and hexuronate, in which the GlcN might be either *N*-acetylated (GlcNAc) or *N*-sulfated (GlcNS), and the hexuronate residues are present as either GlcA or the C-5 epimer, IdoA. Ester *O*-sulfations are principally at the C-2 position of hexuronate (GlcA or IdoA) and the C-6 position of the GlcNS [4,5]. GAGs, except HA, are normally present in the form of proteoglycans (PGs), in which multiple GAGs are covalently attached to a core protein [1,6,7]. Heparin is commercially produced from animal tissues (pig or bovine intestinal mucosa, bovine lung, etc.) and it is clinically used as an antithrombotic drug. Heparin is confined to mast cells, where it is stored in cytoplasmic granules in intact tissue [8,9]. In contrast, HS is ubiquitously distributed on cell surfaces and in the extracellular matrix (ECM) [10,11].

Heparin/HS are implicated in cell adhesion, recognition, migration, and the regulation of various enzymatic activities, as well as their well-known anticoagulant action [11,12,13,14,15]. Most of the biological functions of heparin/HS depend upon the binding of various functional proteins, mediated by specific domains with distinct saccharide sequences [9,16]. For example, the interaction of heparin/HS with fibroblast growth factor (FGF)-1 and FGF-2 requires different saccharide sequences with different combinations of sulfate groups [17,18,19,20].

Polysaccharides that are extracted from brown marine algae, fucoidans, represent a source of marine compounds with potential applications in medicine as naturally occurring heparinoids. Fucoidans are a sub-group of heparinoids that have been proposed as alternative anticoagulants to heparin. Fucoidans are highly sulfated polysaccharides (30–60%), like heparin, but they contain neither *N*-acetylated nor *N*-sulfated groups. Instead, the polysaccharide is primarily composed of 4-sulfated 1,2-linked α-l-fucose with branching or a sulfate group at C-3. Fucoidans have been reported to have anti-aggregation of platelets, and anti-thrombotic, anti-infective and anti-inflammatory activities [21,22,23]. The low hemorrhagic effects of fucoidans as compared to heparin are due to their low anti-aggregation effect [24,25].

Chitin is the major organic component of the exoskeleton of crabs, shrimps, and insects and it is a (1→4 linked) co-polymer of *N*-acetyl-glucosamine units. Chitosan is a product obtained from the de-*N*-acetylation of chitin in the presence of hot alkali [26]. Chitosan interacts with FGF-2 and protects it from inactivation [27]. A chemically sulfonated chitosan, as semi-synthetic heparinoids, has structural and functional similarities to heparin [28]. Chemically, sulfonated dextran (dextran sulfate) has low anticoagulant activity, but high lipoprotein-releasing activity [29]. The treatment of capsular K5 polysaccharide from *Escherichia coli* with mild acid to remove branches affords a (1→4 linked) copolymer of GlcNAc and GlcA [30,31]. New chemical-enzymatic technologies that are based on the modification of bacterial capsular K5 polysaccharides have provided a number of semi-synthetic heparinoids with different biological activities. Two families of sulfated compounds that differ in their hexuronate content have been synthesized while using these technologies. The first group contains only GlcA, whereas the second group contains approximately 50% IdoA following epimerization by immobilized recombinant C5 epimerase [32,33]. This has led to the development of various anticoagulant and non-anticoagulant K5 derivatives following specific ester *O*-sulfations that were endowed with different—and sometimes highly specific—antitumor, antiviral, and/or anti-inflammatory activities [32,33].

The above-mentioned activities of heparin-binding cytokines occur in the ECM through specific non-covalent interactions with, for example, ECM receptor molecules and PGs in which multiple GAGs are covalently attached [34]. Those localized interactions have inspired the development of biomaterials that enhance and regulate the heparin-binding cytokine activities for practical applications [35,36,37]. For example, biomaterials that are modified with heparinoids may exhibit increased stability and controlled release and activation. In addition, polyelectrolytes, such as heparinoids in the ECM, retain heparin-binding cytokines at the cell-material interface via specific interactions [38,39]. Herein, we review the structures of heparin/HS, and biological activities and therapeutic potential of heparinoids. Heparin/HS function to localize and control heparin-binding cytokine activity, as do various heparin/HS-based biomaterials, such as heparin-carrying polystyrene, heparinoid-containing hydrocolloids, polyelectrolyte complex nano/micro-particles (N/MPs), and heparin-coated devices exhibiting the multivalent and cluster effects that result from specific sulfated sequences in heparin/HS. In addition, we highlight our studies while using heparinoid-based biomaterials in heparin-binding cytokine delivery systems.

## 2. Structures of Heparin/HS

### 2.1. Compositional Structures of Heparin and HS

Heparin/HS, which are major groups in heparinoids, are synthesized as PGs, which consist of polysaccharide chains that are covalently bound to a protein core. A single protein, serglycin, is the protein constituent of heparin-PGs in connective tissue mast cells, whereas mucosal mast cells and activated macrophages contain oversulfated chondroitin sulfate [9,23,40]. In contrast, HS can be conjugated onto a variety of proteins with different spatial distributions, e.g., perlecan in the extracellular matrix, and cell-surface associated syndecans with transmembrane core proteins and glypicans that are associated with the plasma membrane via a glycosyl–phosphatidyl–inositol anchor [10,23,41,42].

**Figure 1:**
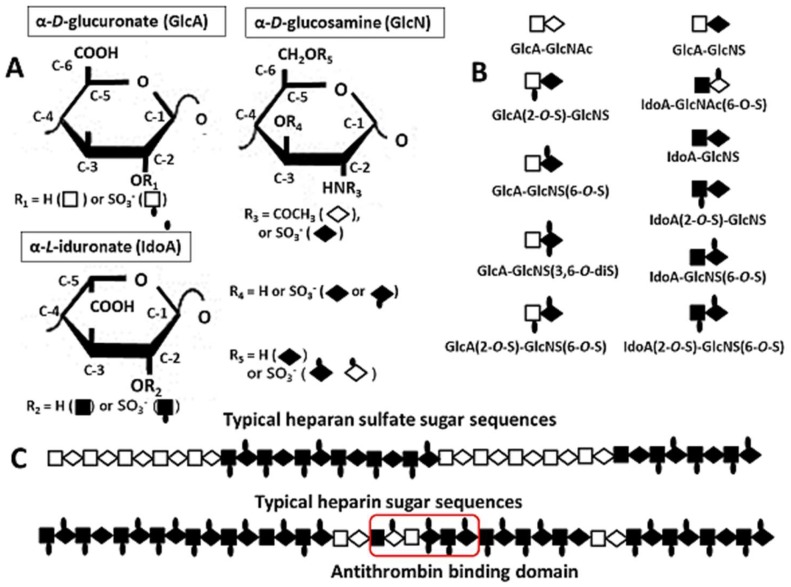
Monosaccharide (**A**) and disaccharide (**B**) units comprising heparin/heparin sulfate (HS), and (**C**) typical heparin sulfate and heparin sugar sequences.

The HS chains influence a multitude of processes in development and homeostasis, due to their ability to interact with a variety of proteins [9,43,44]. Such interactions involve basic amino acid residues and negatively charged carboxyl and sulfate groups along the HS chains mediate them. Heparin and HS both basically consist of a disaccharide repeat of (1→4 linked) α-d-glucosamine and hexuronate, in which the glucosamine residues may be either *N*-acetylated (GlcNAc) or *N*-sulfated (GlcNS), and the hexuronate residues in heparin/HS are present as either β-d-glucuronate (GlcA) or the C-5 epimer, α-l-iduronate (IdoA). Ester *O*-sulfations, principally at the C-2 position of hexuronate (GlcA or IdoA) and the C-6 position of the GlcNS, but also rarely at the C-2 position of GlcNS and the C-3 position of GlcA, add notable charge density and structural complexity to the polysaccharide chains (Figure 1A) [5,45]. Figure 1B shows typical disaccharide sequences that were found in heparin and HS.

The carbohydrate composition for heparin and heparan sulfate (HS) is similar, but it differs in monosaccharide ratios and sulfation pattern distribution. Structural differences between heparin and HS result from differences in their IdoA, and *N*- and *O*-sulfate content. Heparin is extensively *N*-sulfated and it is rich in IdoA and *O*-sulfate groups, whereas HS contains more *N*-acetylated regions [5,8,46]. In general, approximately 80% of the α-d-glucosamine residues in typical commercial heparin are *N*-sulfated, and there is a higher content of *O*-sulfate than of *N*-sulfate. In addition, approximately 70% of the hexuronate in heparin is IdoA, and more than 50% of the disaccharide in heparin is usually trisulfated (IdoA(2-*O*-S)–GlcNS(6-*O*-S)). Furthermore, native heparin contains a GlcNAc (6-*O*-S)–GlcA–GlcNS (3,6-di*O*-S)–IdoA (2-*O*-S)–GlcNS (6-*O*-S) sequence, which is well known as an antithrombin binding domain [47]. In contrast, typically fewer than 50% of glucosamine residues in HS are *N*-sulfated, and the content of *O*-sulfate is lower than that of *N*-sulfate, although there are large differences in HS produced by various cell types.

However, the above distinctions only serve to define the two families of polysaccharides that are composed of the same repeating disaccharide units (i.e., heparan sulfate and heparin sugar sequences) (Figure 1C) [5,46,48]. The molecular design of HS appears to be well adapted for playing a fundamental role in various cellular activities. HS is an ordered polymeric structure in which sulfated sugar residues are clustered in a series of short domains that are widely separated by relatively long regions with low sulfate content [8,16,46]. The glucosamine residues in the highly sulfated clusters are highly *N*-sulfated, and most of the various *O*-sulfates and IdoA residues are present in these domains. However, the trisulfated disaccharide IdoA(2-*O*-S)–GlcNS(6-*O*-S) that is enriched in heparin is a minor component of the highly sulfated regions in HS, and the disulfated disaccharide IdoA(2-*O*-S)–GlcNS is the major disaccharide. The domain organization of HS is a characteristic feature that distinguishes it from heparin (Figure 1C) [9,11,12,48].

### 2.2. Heparin-Based Chemically Modified Sulfated Polysaccharides and Oligosaccharides from Heparin

It is difficult to prepare a large enough amount of the highly sulfated sequences, although the isolation of a highly sulfated sequence from HS responsible for a specific biological activity is one way to establish relationships between structure and function. An alternative approach is to prepare a series of structurally modified oligosaccharides and determine the effects of these structural changes on biological activity. All of the sulfate groups in heparin can be modified to introduce structural changes. Several studies of such heparin molecules have included procedures, such as *N*-desulfation (*N*-DS), 2-*O*-DS [49] and 6-*O*-DS [50,51,52], *N*-deacetylation/sulfation [53,54,55,56], and carboxyl reduction [57]. These modification procedures have been useful in obtaining oligosaccharides with altered biological properties. Furthermore, binding studies of the modified heparinoids to various heparin-binding proteins have revealed several structural features that are involved in binding.

*N*-sulfate groups of heparin can be selectively removed by solvolysis performed by heating the pyridium salt of heparin in dimethyl sulfoxide containing a small amount of water [52,53,54]. When the reaction is performed at 50 °C for a short period of time, almost all of the *N*-sulfate groups are removed, which leaves the other structural features unmodified. A modified solvolytic procedure used for the *N*-DS of heparin can also be applied to 6-*O*-DS. The rates of DS decrease in the order *N*-sulfate > 6-*O*-sulfate ≫ 2-*O*-sulfat when heparin is heated in dimethyl sulfoxide containing a small amount of water at 90 °C [52,53,54]. Most of the 6-*O*-sulfates can be removed while a high proportion of the 2-*O*-sulfates remains, since 6-*O*-DS occurs more rapidly than 2-*O*-DS. Following the reaction, the intermediates can be converted into 6-*O*-DS heparin by the re-*N*-sulfation of *N*-DS glucosamine residues by treatment with a trimethylamine–sulfur trioxide complex in alkaline (pH 9) aqueous media [52]. Another method for specific 6-*O*-DS involves the treatment of heparin (pyridinium salts) with *N*-methyltrimethylsilyl-trifluoroacetamide, which results in specific 6-*O*-DS without detectable depolymerization or other chemical changes [51,52]. Similarly, the complete drying of heparin with various concentrations of NaOH by lyophilization causes specific 2-*O*-DS of hexuronate [49].

The degree of conversion in these *N*- and *O*-DS reactions can be controlled, which permits the preparation of a range of partially modified heparins. Conversion can be controlled by limiting the reaction time or the amounts of reactants consumed in the reaction, or by modifying the reaction conditions [49,51]. These specific and controlled DS reactions result in the formation of unique heparin/HS structures that may offer further possibilities for polymer modification.

### 2.3. Size- and Structure-Defined Oligosaccharides from Heparin and their Affinities for and Activation of FGF

The structural variability of heparin/HS makes it difficult to identify the cytokine-binding domains of a heparin without converting the polymeric heparin to oligosaccharides. Heparins can be partially cleaved while using nitrous acid, heparin lysate, or other methods [58]. All of the cleavage methods yield mixtures containing various oligosaccharide species that vary in both size and structure [58]. Thus, an initial experiment should be conducted to identify the cleavage method that gives the maximum yield of the desired oligosaccharides.

**Figure 2:**
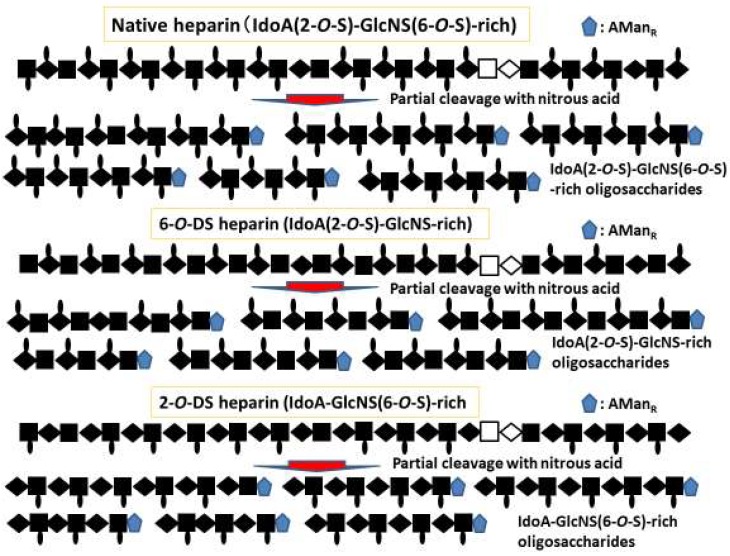
Preparation of size- and structure-defined oligosaccharides from native, 2-*O*-desulfation (DS) and 6-*O*-DS heparins.

A library of size- and structure-defined oligosaccharides was prepared from intact heparin, 2-*O*-DS heparin, and 6-*O*-DS heparin by partial depolymerization with nitrous acid at pH 3 for 10 min., where 2,5-anhydromannitol residues, abbreviated as AMan_R_, were generated at reducing ends (Figure 2) [58]. The resulting oligosaccharides were separated according to size by gel-filtration, and then further fractionated by ion-exchange chromatography to separate them based on their charges. The obtained 6-mers, 8-mers, 10-mers, and 12-mers were enriched in IdoA (2-*O*-S)–GlcNS (6-*O*-S), IdoA–GlcNS (6-*O*-S), and IdoA (2-*O*-S)–GlcNS disaccharide sequences (≧80%). These oligosaccharides were then evaluated for their binding affinities to FGFs and their ability to promote biological activity (Figure 2) [16,58].

Oligosaccharides derived from chemically modified heparins bind to both FGF-1 and FGF-2, with different affinities. Our structural studies using selectively modified 2-*O*- and 6-*O*-DS heparins suggested that the structural requirements for heparin and HS to bind to FGF-1 are different from those for binding to FGF-2 [20,58,59]. For example, the chlorate-treated A31 cells do not produce endogenous sulfated heparan sulfate proteoglycan (HSPG) and intact heparin can restore the mitogenic activities of both FGF-1 and FGF-2 in these cells. The partial 2-*O*-DS of heparin decreases the ability to restore the mitogenic activities of both FGF-1 and FGF-2, and 75% or higher 2-*O*-DS completely abolishes this ability [49]. Similarly, partial 6-*O*-DS of heparin decreases the ability to restore the mitogenic activity of FGF-1, and 62.2% or higher 6-*O*-DS results in the complete loss of mitogenic ability [51]. In contrast, partial 6-*O*-DS up to 66.8% significantly decreased the ability to restore FGF-2 activity. Thus, a high content of 6-*O*-sulfate groups in heparin/HS, in addition to a high content of 2-*O*-sulfate and *N*-sulfate, is required for the activation of FGF-1, but not for FGF-2 [49,51]. Selectively *O*-desulfated heparin was applied to affinity column-immobilized FGF-1 or FGF-2 and eluted while using a discontinuous gradient of NaCl. The salt concentration that was required for complete elution from both columns was dependent on the size and specific structure of the modified heparin [20,52,58].

In general, smaller oligosaccharides (2-mers and 4-mers) from the modified heparins show little affinity for either FGF-1 or FGF-2, whereas the binding affinities of 6-mers, 8-mers, 10-mers, and 12-mers for both FGF-1 and FGF-2 were dependent on the specific structure. Furthermore, 10-mers and 12-mers that were enriched in IdoA (2-*O*-S)–GlcNS (6-*O*-S) disaccharide sequences exhibited high affinities and activations for both FGF-1 and FGF-2, whereas the same-sized oligosaccharides that were enriched in IdoA (2-*O*-S)–GlcNS disaccharide sequences had a weaker affinity to FGF-1, but not FGF-2, than unmodified heparin [17,18]. It should be pointed out that the 6-*O*-sulfate groups of GlcNS residues of large oligosaccharides (10-mers or 12-mers) strongly influence the interaction with FGF-1.

The formation of ternary complexes with heparin/HS, FGF, and FGF-receptors (FGFR) cause the mitogenic activities of FGF-1 and FGF-2 [14,59,60,61,62]. In these complexes, heparin oligosaccharides aid the association of heparin-binding cytokines and their receptors, allowing for functional contacts that promote signaling. In contrast, many proteins, such as FGF-1 and FGF-2, exist or self-assemble into homodimers or multimers in their active states, and these structures are often required for protein activity [61,62]. The common binding motifs required for binding to FGF-1 and FGF-2 were shown to be IdoA (2-*O*-S)–GlcNS (6-*O*-S) disaccharide sequences while using a library of heparin-derived oligosaccharides [58,62,63,64,65]. Furthermore, 6-mers and 8-mers were sufficient for binding FGF-1 and FGF-2, but 10-mers or larger oligosaccharides were required for biological activity [14,58,62,63,64,65]. As 6-mers and 8-mers can only bind to one FGF molecule, they may be unable to promote FGF dimerization.

## 3. Interaction of Heparin/HS with Heparin-Binding Cytokines

Many biological activities of heparin result from its binding to heparin-binding cytokines and its modulation of their activities. These interactions are often very specific: for example, heparin’s anticoagulant activity primarily results from binding antithrombin (AT) at a discrete pentasaccharide sequence that contains a 3-*O*-sulfated glucosamine residue (GlcNAc(6-*O*-S)–GlcA–GlcNS (3,6-di*O*-S)–IdoA (2-*O*-S)–GlcNS (6-*O*-S)) [8,47]. The pentasaccharide was first suggested as that possessing the highest affinity under the experimental conditions that were employed (elution in high salt from the affinity column), which seemed likely to have been selective for highly charged species [47,66,67]. The pentasaccharide sequence within the heparin has tended to be viewed as the unique binding structure [68]. Subsequent evidence has emerged suggesting that net charge plays a significant role in the affinity of heparin for AT while the pentasaccharide sequence binds AT with high affinity and activates AT, and that the 3-*O*-sulfated group in the central glucosamine unit of the pentasaccharide is not essential for activating AT [48,69]. In fact, other types of carbohydrate structures have also been identified that can fulfill the structural requirements of AT binding [69], and a proposal has been made that the stabilization of AT is the key determinant of its activity [48].

A large number of cytokines can be classified as heparin-binding proteins (Table 1). Many functional properties of heparin/HS are ascribed to interactions between the polysaccharides and heparin-binding cytokines. Those interactions generally depend on the presence of specific highly sulfated regions in HS chains [9,12,15,16]. The FGF family (such as FGF-1, FGF-2, and FGF-4) [20,70,71,72,73], platelet-derived growth factor (PDGF) [74,75], hepatocyte growth factor (HGF) [76,77,78], vascular endothelial growth factor (VEGF) [79,80,81], transforming growth factors ((TGF)-β1 [82,83,84] and TGF-β2 [82,83]), midkine (MK) [85,86], interleukins ((IL)-2 [87], IL-6 [88], IL-8 [89], IL-10 [90], and IL-12 [91,92]), platelet factor (PF)-4 [93,94], interferon (IFN)-γ [95,96], granulocyte/macrophage-colony stimulating factor (GM-CSF) [97,98], heparin-binding epidermal growth factor (HB-EGF) [99], monocyte chemotactic protein-1 (MCP-1) [100,101], stem cell factor (SCF) [102], and macrophage inflammatory proteins ((MIP)-1α, [103] and MIP-1β [104]) (Table 1) are included as classes and examples of heparin-binding cytokines.

Early work attempted to identify the unique sequences that are responsible for interaction with heparin-binding cytokines, again employing affinity chromatography followed by elution with a salt gradient (e.g., in the case of FGF-1 and FGF-2) [49,58,105,106], although it was realized that highly sulfated sequences, such as enriched IdoA (2-*O*-S)–GlcNS (6-*O*-S) disaccharide sequences, could exert affinity for many heparin-binding cytokines and their effects. Interpreting these results as providing evidence for preferred binding sequences [106,107] could lead to the potential argument that biological activity predominantly resides in the highly sulfated domains of HS. In addition, surface plasma resonance (SPR) has been utilized to measure the binding kinetics and affinities of various heparin-binding cytokines with heparin while using SPR chips [63,107]. Competitive SPR studies using different lengths of heparin-derived oligosaccharides and different chemically modified heparins were conducted to determine the size dependence and effect of each sulfated group substitution on their interaction.

While the pentasaccharide sequence, which includes the 3-*O*-sulfatedgroup in the central glucosamine unit, undoubtedly binds AT with high affinity and activates it, as described previously, subsequent evidence has emerged that net charge plays a significant role in the affinity of heparin for AT and to activate it [69]. The interactions of heparin/HS with heparin-binding cytokines generally involve both ionic and hydrogen bonding interactions [69,108,109,110]. Arginine and lysine in the proteins are positively charged (basic) amino acids, and hydrogen bonding interactions can involve basic and other polar amino acids (Asn, Gln, Ser, etc.). Typically, ionic and hydrogen bonding residues lie in a spatially tight array positioned on the surface or in a shallow binding pocket of heparin-binding proteins [46,69,108,109,110]. For example, X-ray crystallography has studied the interactions between FGF-2 and heparin-derived oligosaccharides (in the case of a hexasaccharide), suggesting interactions between asparagine and lysine (Asp 28, Asp 102, Lys 27, Lys 126, Lys 136) and glutamine (Gln 135) residues with the oligosaccharide [111].

The conventional concept of cytokines is that they are diffusible and/or mobile factors that act in the solution. However, many cytokines can function in a non-diffusible fashion when immobilized on either the cell surface or the ECM by binding HSPG. HSPG is known to immobilize heparin-binding cytokines, thereby regulating biological functions, such as cell growth, migration, and adhesion [112]. Almost all of the cytokines described in Table 1 exhibit stronger binding to larger oligosaccharides that are composed of trisulfated disaccharide units (IdoA (2-*O*-S)–GlcNS (6-*O*-S)). In fact, the HS chain of endothelial cell proteoglycan can be defined as a copolymer that contains heparin regions in its structure [113]. Furthermore, the very high density and clustering structure of HS chains in HSPG may more strongly interact with heparin-binding cytokines [114,115]. Thus, it is very useful to create HSPG mimics that are composed of a high density and clustering structure of highly sulfated heparin-like domains.

## 4. Non-Anticoagulant (NAC)-Heparin Carrying Polystyrene (NAC-HCPS)

### 4.1. Synthesis of NAC-Heparin and its Applications

**Figure 3:**
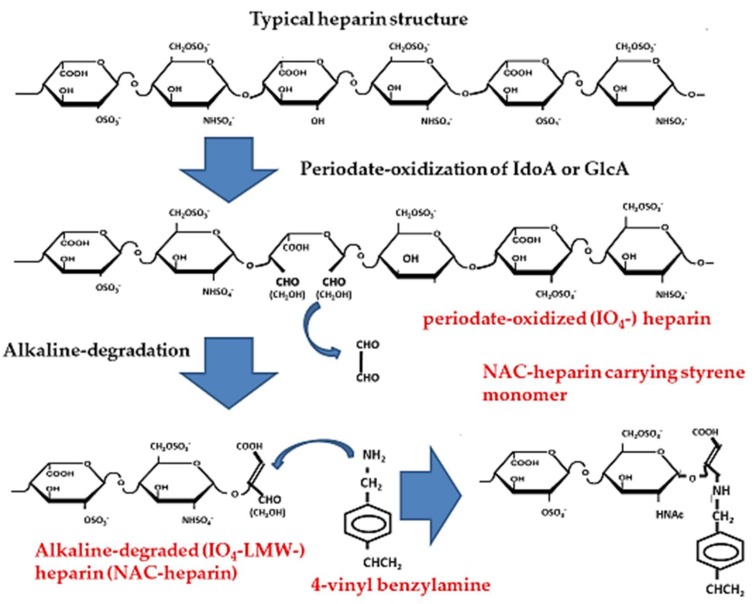
Preparation of periodate-oxidized (IO_4_^−^), alkaline-degraded (IO_4_^−^ low-molecular-weight (LMW))-heparin as non-anticoagulant (NAC)-heparin, and NAC-heparin-carrying styrene monomer.

Heparins are most commonly used as an anticoagulant in injectable solutions for a variety of indications. They specifically interact with functional proteins with high affinity, which include heparin-binding cytokines, ECM components, and adhesion molecules [5,109]. Indeed, heparin is a therapeutic agent for various pathological conditions that involves functional proteins; however, high-dose heparin cannot be used, because of the excessive risk of bleeding [5,109,116]. Non-anticoagulant (NAC)-heparin can be obtained by removing a specific sequence (GlcNAc (6-*O*-S)–GlcA–GlcNS (3,6-di*O*-S)–IdoA (2-*O*-S)–GlcNS (6-*O*-S)) from unfractionated (native) heparin [66,67,68], for example, by specific modifications, such as *N*-desulfation/acetylation, 2-*O*-desulfation, and 6-*O*-desulfation. However, those modifications substantially reduce various physiological activities, as well as the anticoagulant activity of native heparin. A modification of this procedure [116] was used to prepare periodate-oxidized (IO_4_^−^), alkaline-degraded (IO_4_^−^ low-molecular-weight (LMW))-heparin, and NAC-heparin (Figure 3) [117,118,119]. The reduced IO_4_^−^ and IO_4_^−^ LMW-heparins lost unsulfated hexuronate (UA; GlcA or IdoA)-containing structures and they were composed of trisulfated disaccharide units (>85% UA (2-*O*-S)–GlcNS (6-*O*-S)). The interaction of the NAC-heparin with 4 vinyl benzylamine resulted in the production of an NAC-heparin carrying monomer (Figure 3).

The loading of the heparin-based drug delivery systems primarily occurs through an electrostatic mechanism between the negatively charged heparinoids and the positively charged molecular cargo. In addition, negatively or non-charged cargo molecules can be loaded via specific interactions between the heparinoids and cargo molecules [120,121]. Biodegradable heparinoid-based hydrogels that contain cytokines as cargo molecules could be a practical drug delivery system [122].

Water-soluble chitosan molecules (CH-LA) at neutral pH values have been prepared by the introduction of lactose. The material is a viscous solution and readily gels upon mixing with heparinoid solution, which results in an injectable hydrogel being formed through polyelectrolytic interactions between heparinoids (negatively charged), such as NAC-heparin [123,124], 6-*O*-desulfated heparin [125], and fucoidan [126] and CH-LA (positively charged). The subcutaneous injection of FGF-2 containing NAC-heparin/CH-LA into the backs of mice or rats induced marked neovascularization and fibrous tissue formation near the injection sites. Furthermore, the controlled release of biologically active FGF-2 from FGF-2 containing NAC-heparin/CH-LA led to the induction of angiogenesis and, possibly, collateral circulation [123,124] (Table 2).

### 4.2. NAC-HCPS and its Applications

**Figure 4:**
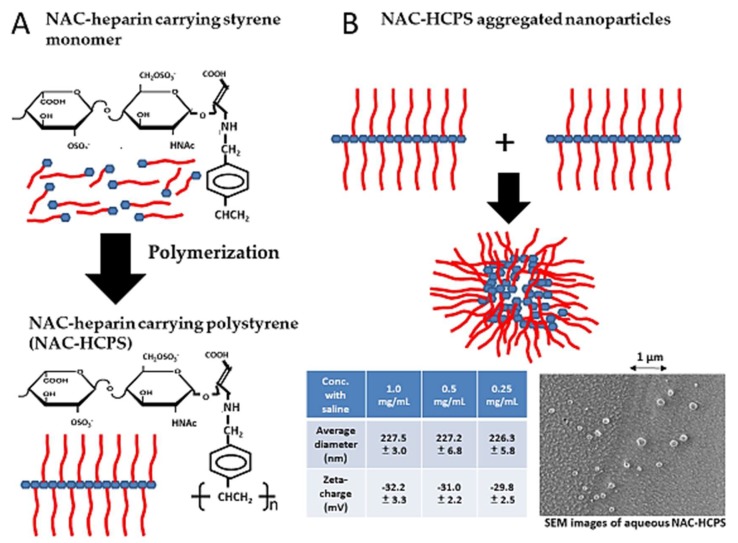
Production of NAC-heparin carrying polystyrene (NAC-HCPS) and NAC-HCPS aggregated nanoparticles. (**A**): Periodate-oxidized (IO_4_^−^), alkaline-degraded (IO_4_^−^ LMW)-heparins are prepared as NAC-heparin, and the interaction of the NAC-heparin with 4 vinyl benzylamine resulted in the production of NAC-heparin carrying monomer. After the polymerization, NAC-heparin carrying polystyrene (NAC-HCPS) was produced. (**B**): The hydrophilic NAC-heparin chains tend to orient toward the outside of the polymer in water, resulting in aggregated nanoparticles with a higher concentration of carbohydrates on the polymer surface.

The simultaneous presentation of multiple copies of biorecognizable saccharide epitopes on an appropriate macromolecular scaffold creates a multivalent display that amplifies the affinity of glycoside-mediated receptor targeting [127]. Indeed, multiple HS and heparin chains are naturally present in HSPG and serglycin (heparin–PG). Saccharide epitopes have been introduced into other forms of heparin/HS-based materials, such as nanoparticles and coatings on various biomedical devices. The drawbacks of the use of heparin and heparin-mimicking materials have been widely studied in light of their therapeutic applications, given the diverse functions of heparin in the body, including anticoagulation, tissue regeneration, anti-inflammation, and protein stabilization [128]. NAC-(IO_4_^−^ LMW)-heparin carrying polystyrene (NAC-HCPS) is a synthetic glycoconjugate that disperses in water and saline. NAC-HCPS has a molecular weight of approximately 80–120 kDa and it comprises approximately ten IO_4_^−^ LMW-heparins linked to a polystyrene core (Figure 4A) [129,130]. The hydrophobic polystyrene core of NAC-HCPS in water might be buried inside the large molecule to form a hydrophobic core sequestered from water [130,131], and NAC-HCPS forms aggregated nanoparticles (Figure 4B) with an average diameter of 220–230 nm and a zeta-charge of about −30 mV. Figure 4B shows an image of NAC-HCPS aggregated nanoparticles obtained while using a cryo-scanning electron microscope (SEM) (JEOL Ltd., Tokyo, Japan).

The synthesized NAC-HCPS showed reduced anticoagulant activity relative to native heparin due to the loss of the antithrombin binding pentasaccharide sequence containing a 3-*O*-sulfated glucosamine residue and enhanced ability to interact with various heparin-binding cytokines, such as FGF, VEGF, and HGF. However, NAC-HCPS strongly inhibited heparin-binding cytokine-induced endothelial cell proliferation in vitro. NAC-HCPS contains a high density of trisulfated disaccharide (IdoA (2-*O*-S)–GlcNS (6-*O*-S)) enriched NAC-heparin chains that are oriented towards the solution. The hydrophilic NAC-heparin chains tend to orient toward the outside of the polymer, which results in a higher concentration of carbohydrates on the polymer surface. An increase in the density of carbohydrate chains greatly enhanced the ability of cell surface receptors to recognize the target [120]. Similarly, enhanced biological activities due to the carbohydrate-clustering effect and immobilization of carbohydrate-clustered PGs have been reported and ascribed to the presence of multiple GAG chains in the core protein [120,130]. The very high density and clustering structure of NAC-heparin chains might not support the overall interaction of heparin-binding cytokines with their receptors to induce mitogenic activities, although NAC-HCPS interacts more strongly with cytokines than NAC-heparins. In fact, NAC-HCPS inhibited angiogenesis and subcutaneous induced tumor growth and metastasis in vivo [131], as well as neointimal proliferation of balloon-injured arteries [132].

Lactose-carrying polystyrene, poly(*N*-*p*-vinyl-benzyl-4-*O*-*D*-gluconamide) (PVLA), was previously developed as a synthetic glycoconjugate that adsorbs to plastic plates and possesses unique properties as a substratum, thereby mediating the interaction with carbohydrate receptors for the primary culture of rat hepatocytes [133]. Similarly, NAC-HCPS is efficiently adsorbed onto plastic surfaces, such as those of tissue culture plates, and heparin-binding cytokines are immobilized on the surface of NAC-HCPS-coated plates [131]. Mouse adipose tissue-derived stromal cells (ADSCs) grew well in low serum and they maintained their multilineage potential for differentiation on NAC-HSPS-coated plates in the presence of FGF-2 [134,135] (Table 2). Thus, NAC-HCPS-coated plates, together with FGF-2 in low-serum media, might be useful for autologous ADSC expansion in clinical cell therapy.

## 5. Heparin-Based Polyelectrolyte Complex Nano/Micro-Particles (N/MPs) and their Applications

### 5.1. Low-Molecular-Weight Heparin/Protamine (LMWH/P) N/MPs for Cytokine Carrier

Mechanisms, critical experimental aspects, and applications of polyelectrolyte complexes (PECs) were comprehensively reviewed [136]. The present review focuses on PEC hydrogels that formed by the chemical interaction of chitosan and crosslinkers [137,138]. Heparinoids, which are conjugated to nano-materials, have been recently investigated for their chemical and biological properties and applications [35,37]. Heparinoid has been conjugated to the surface of nanoparticles (NPs), such as magnetic [139] and metallic NPs [140], heparin-coated nanoparticles coupled to hemoglobin [141], and biopolymers [142]. Furthermore, Syndecan-4 proteoliposomes enhance FGF-2-induced proliferation, migration, and neovascularization of ischemic muscles [143]. When biological molecules, such as functional proteins and DNA, are incorporated on or within NPs, they provide novel and enhanced activities with potential applications in therapeutics, biosensors, imaging, and drug delivery [35,37,144]. Those biomolecules can be passivated on NPs, thus improving their biocompatibility.

Electrostatic interactions between oppositely charged polyelectrolytes, such as low-molecular-weight heparin (LMWH) (MW: approximately 5000 Da) and protamine, generate PECs (Figure 6A). When this interaction occurs in non-equivalent ratios, nonstoichiometric PECs are produced, which causes each PEC particle to carry an excess charge [37,145,146]. Proteins interact with both synthetic and natural PECs [26]. Heparin is useful as a therapeutic agent in various pathological conditions that involve heparin-binding cytokines. However, high-dose heparin cannot be used because of the excessive risk of bleeding [117]. In contrast, LMWH offers pharmacological and practical advantages when compared with heparin. The lower protein-binding activity of LMWH produces a low, stable, and predictable anticoagulant response, thereby bypassing the need for laboratory monitoring of drug levels to adjust the dosage [117]. In addition, one or two subcutaneous injections per day are sufficient for maintaining therapeutic concentrations because of its longer plasma half-life [117]. Protamine, which is a purified mixture of proteins obtained from fish sperm, neutralizes heparin and LMWH by forming a stable complex that lacks anticoagulant activity [147]. Protamine is also clinically employed to reverse the anticoagulant activity of heparin following cardiopulmonary bypass as well as in cases of heparin-induced bleeding [148].

**Figure 5:**
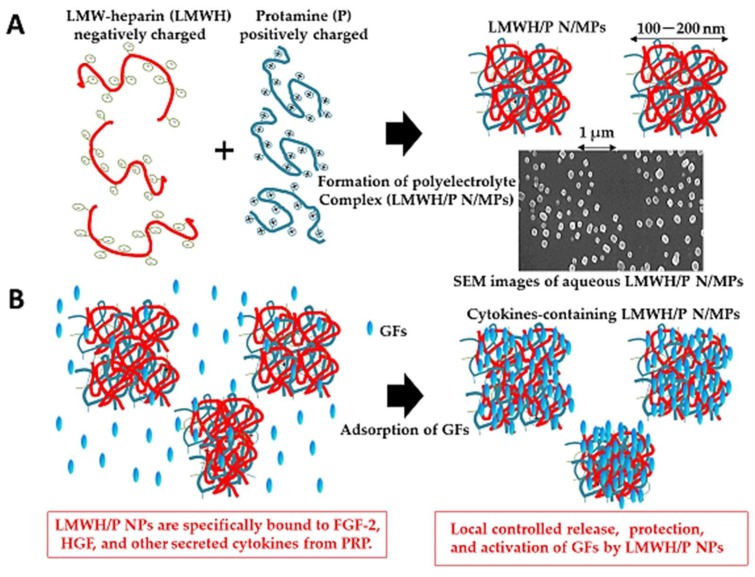
Generation of cytokine-containing low-molecular-weight heparin and protamine nano/micro-particles (LMWH/P N/MPs) as polyelectrolyte complexes (PECs). (**A**): PECs are generated by electrostatic interactions between oppositely charged LMWH and protamine as nano/micro-particles (N/MPs). (**B**): Production of growth factors-containing LMWH/P N/MPs as PECs. Heparin-binding cytokines, such as fibroblast growth factor-2 (FGF-2), hepatocyte growth factor (HGF), and cytokines from platelet-rich plasma (PRP) were bound to the surface of LMWH/P N/MP.

Round Low-molecular-weight heparin and protamine nano/micro-particles (LMWH/P N/MPs) have previously been prepared as PECs by mixing LMWH with protamine at a ratio of 6:4 (Figure 5A) [37,149,150]. LMWH/P N/MPs are 50–200 nm in diameter and they have a zeta charge of −25 to −30 mV. LMWH/P N/MPs specifically bind to heparin-binding growth factors (GFs) such as FGF-2 [149,150], HGF [151], and other cytokines that are secreted from platelet-rich plasma (PRP) (Figure 5B) [152,153], and they can stabilize, control the release, and activate those cytokines [37,152].

Those GFs, and cytokines from PRP-containing LMWH/P N/MPs induce neovascularization and result in collateral blood vessel formation [150,151,153,154,155,156]. LMWH/P N/MPs can be retained on the cell surfaces and matrixes in various tissues in vivo to control the release of cytokine-containing LMWH/P N/MPs, and they can protect and activate cytokine-containing LMWH/P N/MPs induced vascularization and fibrous tissue formation by stabilizing, activating, and gradually releasing their cargo of cytokines [150,151,152,153,154,155,156]. Thus, FGF-2 and cytokines from PRP-containing LMWH/P N/MPs also have stimulatory effects on human hair regrowth [157,158] and the enhancement of mitomycin C-treated [159] and radiation-induced healing-impaired wound repair [160]. Furthermore, skin flap necrosis was prevented [161] and topical pre-injection of cytokines from PRP-containing LMWH/P N/MPs promoted epithelialization and angiogenesis in split-thickness skin graft donor sites [162]. Furthermore, the survival and healing of wounds in a crush syndrome model of rat were promoted by the injection of FGF-2-containing LMWH/P N/MPs [163] (Table 3). However, it is necessary to understand intramuscular pharmacokinetics and guide the local drug delivery for effective local intramuscular injection [164].

### 5.2. LMWH/P N/MPs for Cell Carrier

**Figure 6:**
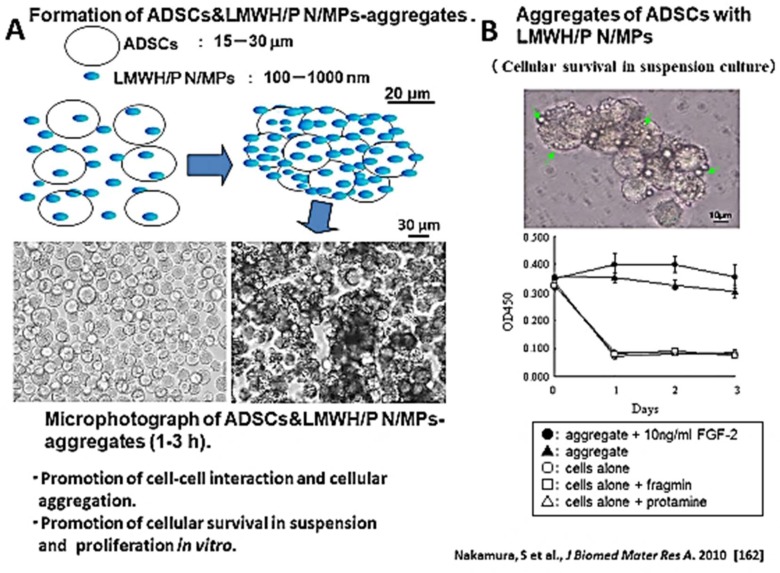
LMWH/P NPs as a cell carrier. (**A**): LMWH/P N/MPs bind to adipose tissue-derived stromal cells (ADSCs) through specific interactions between the LMWH/P N/MPs and cell surface heparin-binding proteins. The interaction of cells with LMWH/P N/MPs resulted in the formation of aggregates comprising cells and LMWH/P N/MPs within 1–3 h. (**B**): These aggregates increased the cellular viability in vitro.

LMWH/P N/MPs bind to various adhesive cell surfaces, including adipose-derived stromal cells (ADSCs) and bone marrow-derived mesenchymal stem cells (BMSCs), as well as tumor cells, through specific interactions between the LMWH/P N/MPs and cell surface heparin-binding proteins [37,165,166,167]. The interaction of cells with LMWH/P N/MPs resulted in the formation of aggregates that comprise cells and LMWH/P N/MPs within a few hours (Figure 6A). These aggregates increased the cellular viability in vitro [165,167]. Injection of these aggregates induced vascularization and fibrous tissue formation in vivo [167]. Furthermore, LMWH/P N/MPs that are used as cell carriers can enhance cell viability in vivo (Figure 6B).

LMWH/P N/MPs efficiently bind to tissue culture plates to act as a coating matrix. The ability of LMWH/P N/MPs to retain heparin-binding cytokines could make them very useful in two-dimensional cell culture [168,169,170]. Human microvascular endothelial cells and human dermal fibroblast cells adhered well to LMWH/P N/MP-coated suspension culture plates [171] and they grew rapidly in low (1–2%) fetal bovine serum (FBS) medium that was supplemented with FGF-2. This approach could allow for the use of low autologous serum (1–2%) for culturing BMSCs and ADSCs [167,168]. CD34+ hematopoietic progenitor cells (CD34+ HCs) that were derived from mouse bone marrow showed higher proliferation on LMWH/P N/MP-coated plates in hematopoietic progenitor growth medium that was supplemented with the appropriate cytokines than on the uncoated plates [170]. Furthermore, ADSCs and BMSCs, as well as other adhesion cells, can also be grown efficiently in three-dimensional (3D) culture while using low human plasma (3%)-DMEM gel containing LMWH/P N/MPs without animal serum [171,172].

Various biomaterials have been used as cell carriers during cell implantations [173,174]. Inbred rat (IR) plasma (IRP)-DMEM gel with LMWH/P N/MPs, the biomaterial that were tested in this study, can carry many IR-ADSCs and also act as a cell carrier in which the cells can grow. It has been reported that many LMWH/P N/MPs can bind to surfaces of ADSCs, and the interaction of ADSCs with LMWH/P N/MPs induces ADSCs and LMWH/P N/MP-aggregate formation, and substantially maintains cell viability for at least three days in suspension-culture conditions [167]. In contrast, it appears that the interaction of IR-ADSCs in IRP-DMEM gel as a three-dimensional (3D) matrix stimulates the growth of IR-ADSCs and generates a 3D network [171,172]. Furthermore, the growth factors that were secreted from IR-ADSCs, as well as the growth factors derived from the IRP, may be retained within the IRP-DMEM gel with LMWH/P N/MPs [175] (Table 4).

We previously applied 3D-cultured IR-ADSCs that were derived from inbred male Fisher 344 rats while using injectable low IRP (3%)-DMEM gel with LMWH/P N/MPs for cell transplantation [175]. Furthermore, IR-ADSCs using IRP (6%)-DMEM gel with LMWH/P N/MPs/FGF-2 were applied to full thickness skin excisions as healing-impaired wounds on the backs of STZ-induced diabetic rats [176]. The wound closures that were treated with IR-ADSCs while using IRP-DMEM gel with LMWH/P N/MPs/FGF-2 were significantly enhanced in post-wounding [176] (Table 4). The histological examination of wounds that were treated with IR-ADSCs while using IRP-DMEM gel with LMWH/P N/MPs/FGF-2 demonstrated significantly advanced epithelialization, capillary formation, and granulation tissue formation.

## 6. Heparinoid-Coated Devices

Research has shown that there are heparin-like molecular structures in microvascular endothelial cells; when the solidification state of blood changes, such endothelial cells will activate [114], and heparanase regulates thrombosis in vascular injury [177]. Therefore, it might be accepted after all as a reasonable method for modifying the abiotic surface with heparinoids to give it high blood compatibility. Heparin solidification has been widely studied on the blood contact surface of cardiopulmonary bypass (CPB) equipment, catheters, vascular stents, and coronary stents [178,179]. Heparin immobilization technologies can be divided into two broad categories: eluting technologies that release heparin from the device, and non-eluting technologies that are intended for permanent covalent immobilization of heparin to the device surface. Release-based approaches are essentially drug delivery systems that can prevent local device related acute thrombosis. The release rate of heparin from the surface might be tailored through physical entrapment or the ionic binding of heparin to the surface. For example, complexes of heparin with branched surfactants bearing quaternary ammonium groups can be deposited on a material surface. Blood contact causes the release of the ionic complex from the surface, but the presence of the surfactant slows the rate of heparin release. Other elution-based technologies have been described in previous reviews [180].

Heparin, as the first compound considered for stent-based delivery, was chosen on the basis of promising tissue culture and animal experiments. However, heparin has failed to stop restenosis clinically. Recently used compounds, such as paclitaxel, are of a different sort, being hydrophobic in nature, and their effects after local release are far more profound. The study suggested that physiological transport forces cause local concentrations to significantly deviate from mean concentrations [181]. In fact, the controlled release of paclitaxel from photocrosslinked chitosan hydrogels inhibited subcutaneous tumor growth in mice [182].

Immobilizing heparin to the device surface is the alternative approach intended to confer long-term surface thrombo-resistance. Heparin can be readily immobilized by different surface conjugation chemistries, by virtue of its strong net negative charge, low pKa due to the abundance of carboxyl and sulfo-groups, and a variety of chemically active functional groups [183]. Each repeating disaccharide unit offers at least one carboxyl and hydroxyl functional group, which might be activated and subsequently attached to a compatible functional group on the target surface. This might be achieved by direct chemical activation of the surface to introduce complementary functional groups for immobilization of heparin, or through the application of an intermediary priming matrix to which the heparin can be covalently linked. For example, the carbodiimide crosslinker EDC has been used to activate carboxyl groups along the heparin chain and then conjugate to amines that were incorporated into the target surface [184]. Periodate oxidation of vicinal diols introduces aldehydes along the heparin chain, which might also be linked to an aminated surface by reductive amination [185].

## 7. Overview

Heparinoids can be used in drug delivery systems and coating devices in the fields of tissue engineering and biotechnology. Heparinoids are associated with various biological processes of heparin-binding cytokines, in addition to their well-known anticoagulant action, and they are implicated in cell adhesion, recognition, migration, and the regulation of enzymatic activities. The use of heparinoids in medical and biotechnological fields often requires adequate chemical modification of the polymers to change their properties and influence their affinities for heparin-binding cytokines. As described in this review, understanding the interaction of native and modified heparin/HS with heparin-binding cytokines could result in the production of valuable heparinoid-based biomaterials for controlled protein delivery.

Many studies have identified the specific binding sequences for different heparin-binding proteins, and some biochemical processes, such as anticoagulation and FGF signaling, only proceed with specific binding sequences, as described in this review. IO_4_^−^ LMW-heparin is simply modified NAC-heparin; when composed of more than 85% trisulfated disaccharide units (IdoA (2-*O*-S)–GlcNS (6-*O*-S)), this modified heparin can interact with almost all functional proteins and affect their biological activities. However, even NAC-heparin polysaccharides often have only weak biological activities in vivo.

Heparin/HS are highly soluble and dispersible in water and, thus, engineering approaches are important to further reinforce their biological activities to improve their in vivo applicability as biomaterials. The practical application of heparin/HS often involves the use of an adequate medium, such as hydrogels, HCPS, or PECs, to retain the formed complexes and the multivalent and cluster effects of the functional sequences in the heparin/HS. The covalent or non-covalent modification of biomaterials with heparin/HS can augment their stability, localization, controlled release, and activation. In addition, assemblies of heparinoids and other polyelectrolytes retain heparin-binding cytokines at the local cell–material interfaces via specific non-covalent interactions. The present review discussed heparin/HS-based biomaterials, such as NAC-HCPS, hydrogels, membranes, and LMWH/P N/MPs, and their functions with respect to their applications as versatile biomaterials. Such heparin/HS-based biomaterials have been shown to be safe and efficacious in vivo for the delivery of a variety of heparin-binding molecules.

## Figures and Tables

**Table 1:** Classes and examples of heparin-binding cytokines.

Full Name (Family)	Abbreviations	Functions	References
Fibroblast growth factor family	FGF-1 FGF-2 FGF-4	Potential effects in the repair and regeneration of tissues and in development.	[20,70,71,72] [20,70,71,72] [20,73]
Platelet-derived growth factor	PDGF-A PDGF-BB	Blood vessel formation, mitogenesis, and proliferation of mesenchymal cells.	[74] [75]
Hepatocyte growth factor	HGF	Cell growth, cell motility, and morphogenesis by activating a tyrosine kinase.	[76,77,78]
Vascular endothelial growth factor	VEGF	Angiogenesis, bone formation, hematopoiesis, wound healing, and development.	[79,80,81]
Transforming growth factor-β family	TGF-β1 TG F-β2	Cell growth, development, homeostasis, and regulation of the immune system.	[82,83,84] [82,83]
Midkines	MK	Development, reproduction, and repair, and in the pathogenesis of inflammatory diseases.	[85,86]
Interleukin family	IL-2, IL-6 IL-8, IL-10 IL-12	Development and differentiation of T and B lymphocytes, and hematopoietic cells.	[87,88] [89,90] [91,92]
Platelet factor-4	PF-4	Chemoattractant for neutrophils and fibroblasts, a role in inflammation and repair.	[93,94]
Interferon-γ	IFN-γ	Antiviral, immunoregulatory, and anti-tumor properties.	[95,96]
Granulocyte/macrophage-colony stimulating factor	GM-CSF	Stimulation of stem cells to produce granulocytes and monocytes.	[97,98]
Heparin-binding epidermal growth factor	HB-EGF	Wound healing, cardiac hypertrophy, and heart development.	[99]
Monocyte chemotactic protein-1	MCP-1	Promotion of recruitment of monocytes and macrophages.	[100,101]
Stem cell factor	SCF	Hematopoiesis, supermagenesis, and melanogenesis.	[102]
Macrophage-inflammatory protein-1	MIP-1α MIP-1β	Activation of granulocytes, which can lead to acute neutrophilic inflammation.	[103] [104]

**Table 2:** Biomedical applications of NAC-heparin and NAC-HCPS as biomaterials.

Applications	Overview	References
Injection of NAC-heparin/CH-LA	Induction of angiogenesis and collateral circulation by subcutaneous injection of FGF-2 containing NAC-heparin/chitosan–lactose (CH-LA)	[123,124]
Inhibition of angiogenesis and tumor metastasis in vivo	NAC-HCPS inhibited angiogenesis and subcutaneous induced tumor growth and metastasis in vivo	[131]
Inhibition of neointimal proliferation of balloon-injured arteries	NAC-HCPS inhibited smooth muscle cell growth in vitro and neointimal proliferation of balloon-injured arteries in vivo	[132]
Substratum for cell cultures	NAC-HCPS is efficiently adsorbed onto plastic surfaces such as those of tissue culture plates, and heparin-binding cytokines are immobilized on the surface of NAC-HCPS-coated plates	[134,135]

**Table 3:** Biomedical applications of cytokine-containing LMWH/P N/MPs as biomaterials.

Applications	Overview	References
Carrier for FGF-2, HGF, and cytokines from platelet-rich plasma	Adsorption, stabilization, controlled release, and activation of FGF-2, HGF, and cytokines from platelet-rich plasma (PRP).	[149,150] (FGF-2) [151] (HGF) [152] (Cytokines from PRP)
Neovascularization	Induction of collateral blood vessel formation in rabbit by FGF-2, HGF, and cytokines from PRP-containing LMWH/P N/MPs.	[150,154,155] (FGF-2) [151] (HGF) [153,156] (Cytokines from PRP)
Hair regrowth	Enhancement of human hair growth by FGF-2 and cytokines from PRP-containing LMWH/P N/MPs.	[157] (FGF-2) [158] (Cytokines from PRP)
Injection of cytokines from PRP into skin for healing-impaired wound	Enhancement of mitomycin C-treated healing-impaired wound by cytokines from PRP-containing LMWH/P N/MPs.	[159]
Injection of cytokines from PRP into skin for healing-impaired wound	Enhancement of radiation-induced healing-impaired wound repair by FGF-2-containing LMWH/P N/MPs.	[160]
Injection of cytokines from PRP into skin for skin flap necrosis	Prevention of skin flap necrosis by topical injection of cytokines from PRP-containing LMWH/P N/MPs.	[161]
Injection of cytokines from PRP into skin for split-thickness skin graft donor sites	Promotion of epithelialization and angiogenesis in split-thickness skin graft donor sites by pre-injection of cytokines from PRP-containing LMWH/P N/MPs.	[162]
Injection of FGF-2 into skin for wounds in crush syndrome	Promotion of survival and healing of wounds in crush syndrome model of rat by injection of FGF-2-containing LMWH/P N/MPs.	[163]

**Table 4:** Biomedical applications of LMWH/P N/MPs as cell carriers.

Applications	Overview	References
Formation of cell aggregates	Formation of cell aggregates by the interaction of cells with LMWH/P N/MPs and increase of cellular viability.	[165] (Tumor cells) [167] (ADSCs)
2D expansion of cells	The ability of LMWH/P N/MPs to retain heparin-binding cytokines. Various cells two-dimensionally expand on those cytokine-coated plates.	[167,168] (ADSCs and BMSCs) [169] (Adhesive cells) [170] (Hematopoietic pro- genitor cells)
3D expansion of cells	Various cells can also be grown efficiently in three-dimensional (3D) culture using low human plasma-DMEM gel containing LMWH/P N/MPs.	[171] (Adhesion cells) [172] (ADSCs and BMSCs)
Transplantation of ADSCs	Transplantation of 3D-cultured IR-ADSCs derived from inbred rats using injectable low IRP (3%)-DMEM gel with LMWH/P N/MPs.	[175]
	Transplantation of 3D-cultured IR-ADSCs derived from inbred rats using injectable IR-ADSCs using IRP (6%)-DMEM gel with LMWH/P N/MPs/FGF-2.	[176]

## References

[1] Kjellen L., Lindahl U. (1991). Proteoglycans: Structure and interaction. Annu. Rev. Biochem..

[2] Gandhi N.S., Mancera R.L. (2008). The structure of glycosaminoglycans and their interactions with proteins. Chem. Biol. Drug Design.

[3] Prydz K. (2015). Determinants of glycosaminoglycan (GAG) strucuture. Biomolecules.

[4] Mirsra S., Hascall V.C., Atanelishvili I., Rodriguez R.M., Markwald R.R., Ghatak S. (2015). Utilization of glycosaminoglycan/proteoglycans as carriers for targeted therapy delivery. Int. J. Cell Biol..

[5] Casu B., Vlodavsky I., Sanderson R.D. (2008). Non-anticoagulant heparins and inhibition of cancer. Pathophysiol. Haematol. Thromb..

[6] Bernfield M., Gotte M., Park P.W., Reizes O., Fitzgerald M.L., Lincecum J., Zako M. (1999). Function of cell surface heparan sulfate proteoglycans. Ann. Rev. Biochem..

[7] Lindahl U., Lidholt K., Spillmann D., Kjellen L. (1994). More to “heparin” than anticoagulation. Thromb. Res..

[8] Lindahl U., Kejellen L. (2013). Pathophysiology of heparan sulfate: Many diseases, few drugs. J. Intern. Med..

[9] Casu B., Lindahl U. (2001). Structure and biological interaction of heparin and heparan sulfate. Adv. Carbohydr. Chem. Biochem..

[10] Murdoch A.D., Dodge G.R., Cohen I., Tuan R.S., Iosso R.V. (1992). Promery structure of human heparansulfate proteoglycan from basement membrane (HSPG2/perlecan). J. Biol. Chem..

[11] Nader H.B., Lopes C.C., Rocha H.A., Santos E.A., Dietrich C.P. (2004). Heparins and heparinoids: Occurrence, structure and mechanism of antithrombotic and hemorrhagic activities. Curr. Pharm. Des..

[12] Lyon M., Gallagher J.T. (1988). Bio-specific sequences and domains in heparan sulphate and regulation of cell growth and adhesion. Matrix Biol..

[13] Coombe D.R., Kett W.C. (2005). Heparan sulfate-protein interactions: Therapeutic potential through structure-function insights. Cell. Mol. Life Sci..

[14] Mohammadi M., Olsen S.K., Ibahimi O.A. (2005). Structural basis for fibroblast growth factor activation. Cytokine Growth Factor Rev..

[15] Lortat-Jacob H. (2009). The molecular basis and functional implications of chemokine interactions with heparan sulfate. Curr. Opin. Struct. Biol..

[16] Ishihara M., Guo Y., Wei Z., Yang Z., Swiedler S.J., Orellana A., Hirschberg C.B. (1993). Regulation of biosynthesis of the basic fibroblast growth factor binding domains of heparan sulfate by heparan sulfate-*N*-deacetylase/*N*-sulfotransferase expression. J. Biol. Chem..

[17] Rapraeger A.C., Krufka A., Olwin B.B. (1991). Requirement of heparan sulfate for bFGF-mediated fibroblast growth and myoblast differentiation. Science.

[18] Yayon A., Klagsbun M., Esko J.D., Leder P., Ornitz D.M. (1991). Cell surface, heparin-like molecules are required for binding of basic fibroblast growth factor to its high affinity receptor. Cell.

[19] Ishihara M., Shaklee P.N., Yang Z., Liang W., Wei Z., Stack R.J. (1994). Structural features in heparin which modulate specific biological activities mediated by basic fibroblast growth factor. Glycobiology.

[20] Ishihara M. (1994). Structural requirements in heparin for binding and activation of FGF-1 and FGF-4 are different from that for FGF-2. Glycobiology.

[21] de Azevedo T.C., Bezerra M.E., Santos M.G.L., Souza L.A., Marques C.T., Benevides N.M., Leite E.L. (2009). Heparinoids algel and anticoagulant, hemorrhage activities and platelet aggregation. Biomed. Pharmacother..

[22] Kariya Y., Watabe S., Kyougashima M., Ishihara M., Ishii T. (1997). Structure of fucose branches fucan in the glycosaminoglycan from the body wall of sea cucumber Stichopus japonicas. Carbohydr. Res..

[23] Rabenstein D.L. (2002). Heparin and heparan sulfate: Structure and function. Nat. Prod. Rep..

[24] Zhu Z., Zhang Q., Chen L., Ren S., Xu P., Tang Y., Luo D. (2010). Higher specificity of the activity of low molecular weight fucoidan for thrombin-induced platelet aggregation. Thromb. Res..

[25] Manne B.K., Getz T.M., Hughes C.E., Alshehri O., Dangelmaier C., Naik U.P., Watson S.P., Kunapuli S.P. (2013). Fucoidan is a novel platelet agonist for the C-type lectin-like receptor 2 (CLEC-2). J. Biol. Chem..

[26] Li J., Cai C., Li J., Li J., Li J., Sun T., Wang L., Wu H., Yu G. (2018). Chitosan-based nanomaterials for drug delivery. Molecules.

[27] Masuoka K., Ishihara M., Asazuma T., Hattori H., Matsui T., Takase B., Kanatani Y., Fujita M., Saitoh Y., Yura H. (2005). The interaction of chitosan with fibroblast growth factor-2 and its protection from inactivation. Biomaterials.

[28] Schatz C., Bionaz A., Lucas M.J., Pichot C., Viton C., Domard A., Delair T. (2005). Formation of polyelectrolyte complex particles from self-complexation of *N*-sulfated chitosan. Biomacromolecules.

[29] Delair T. (2011). Colloidal polyelectrolyte complexes of chitosan and dextran sulfate towards versatile nanocarriers of bioactive molecules. Eur. J. Pharm. Biopharm..

[30] Wang Z., Ly M., Zhong W., Suen A., Hickey A.M., Dordick J.S., Linhardt R.J. (2010). *E. coli* K5 fermentation and the preparation of heparosan, a bioengineered heparin precursor. Biotechnol. Bioeng..

[31] Higashi K., Ly M., Wang Z., Masuko S., Bhaskar U., Sterner E., Zhang F., Toida T., Dordick J.S., Linhardt R.J. (2011). Controlled photochemical depolymerization of K5 heparosan, a bioengineered heparin precursor. Carbohydr. Polym..

[32] Joice A., Raman K., Mencio C., Quintero M.V., Brown S., Nguyen T.K., Kuberan B. (2015). Enzymatic synthesis of heparin sulfate and heparin. Methods Mol. Biol..

[33] Oreste P., Zoppetti G. (2012). Semi-synthetic heparinoids. Handb. Exp. Pharmacol..

[34] Schonherr E., Hausser H.-J. (2000). Extracellular matrix and cytokines: A functional unit. Dev. Immunol..

[35] Kemp M.M., Linhardt R.J. (2010). Heparin-based nanoparticles. WIREs Nanomed. Nanobiotechnol..

[36] Jiao Y., Ubrich N., Marchand-Arvier M., Vigneron C., Hoffman M., Maincent P. (2002). In vitro and in vivo evaluation of oral heparin-loaded polymeric nanoparticles in rabbits. Circulation.

[37] Ishihara M., Kishimoto S., Takikawa M., Hattori H., Nakamura S., Shimizu M. (2015). Biomedical application of low molecular weight heparin/protamine micro/nanoparticles as cell- and growth factor-carriers and coating matrix. Int. J. Mol. Sci..

[38] Berth G., Voigh A., Dautzenberg H., Donath E., Mohwald H. (2002). Polyelectrolyte complex and layer-by layer capsules from chitosan/chitosan sulfate. Biomacromolecules.

[39] Sotiropoulou M., Bokias G., Staikos G. (2005). Water-soluble complexes through coulombic interactions between bovine serum albumin and anionic polyelectrolytes grafted with hydrophilic nonionic side chains. Biomacromolecules.

[40] Kolset S.O., Tveit H. (2008). Serglycin-structure and biology. Cell Mol. Life Sci..

[41] Belting M. (2003). Heparan sulfate proteoglycan as a plasma membrane carrier. Trends Biochem. Sci..

[42] Christianson H.C., Belting M. (2014). Heparan sulfate proteoglycan as a cell-surface endocytosis receptor. Matrix Biol..

[43] Sarrazin S., Lamanna W.C., Esko J.D. (2011). Heparan sulfate proteoglycans. Cold Spring Harb. Perspect Biol..

[44] Esko J.D., Selleck S.B. (2002). Order out of chaos, assembly of ligand binding sites in heparin sulfate. Annu. Rev. Biochem..

[45] Miller T., Goude M.C., McDevitt T.C., Temenoff J.S. (2014). Molecular engineering of glycosaminoglycan chemistry for biomolecule delivery. Acta Biomater..

[46] Gallagher J.T., Turnbull J.E., Lyon M. (1992). Patterns of sulfation in heparan sulphate polymorphism based on a common structural theme. Int. J. Biochem..

[47] Lindahl U., Backstrom G., Thunberg L., Leder I.G. (1980). Evidence for 3-*O*-sulfated d-glucosamine residue in the antithrombin-binding sequence of heparin. Proc. Natl. Acad. Sci. USA.

[48] Meneghetti M.C.Z., Hughes A.J., Rudd T.R., Nader H.B., Powell A.K., Yates E.A., Lima M.A. (2015). Heparan sulfate and heparin interactions with proteins. Interface.

[49] Ishihara M., Kariya Y., Kikuchi H., Minamisawa T., Yoshida K. (1997). Importance of 2-*O*-sulfate groups of uronate residues in heparin for activation of FGF-1 and FGF-2. J. Biochem..

[50] Imberty A., Lortat-Jacob H., Perez S. (2007). Structural view of glycosaminoglycan-protein interactions. Carbohydr. Res..

[51] Ishihara M., Takano R., Kanda T., Hayashi K., Hara S., Kikuchi H., Yoshida K. (1995). Importance of 6-*O*-sulfate groups of glucosamine residues in heparin for activation of FGF-1 and FGF-2. J. Biochem..

[52] Kariya Y., Kyogashima M., Suzuki K., Isomura T., Sakamoto T., Horie K., Ishihara M., Takano R., Kamei K., Hara S. (2000). Preparation of completely 6-*O*-desulfated heparin and its ability to enhance activity of basic fibroblast growth factor. J. Biol. Chem..

[53] Inoue Y., Nagasawa K. (1976). Selective *N*-desulfation of heparin with dimethyl sulfoxide containing water or methanol. Carbohydr. Res..

[54] Nagasawa K., Inoue Y., Kamata T. (1977). Solvolytic desulfation of glucosaminoglycuronan sulfates with dimethyl sulfoxide containing water or methanol. Carbohydr. Res..

[55] Lundin L., Larsson H., Kreuger J., Kanda S., Lindahl U., Salvimirta M., Claesson-Welsh L. (2009). Selectively desulfated heparin inhibits fibroblast growth factor-induced mitogeneity and angiogenesis. J. Biol. Chem..

[56] Raman K., Kuberan B., Arungundram S. (2015). Chemical modification of heparin and heparosan. Methods Mol. Biol..

[57] Garg H.G., Mrabat H., Yu L., Freeman C., Li B., Zhang F., Linhardt R.J., Hales C.A. (2010). Effect of carboxyl-reduced heparin on the growth inhibition of bovine pulmonary artery smooth muscle cells. Carbohydr. Res..

[58] Ishihara M., Tyrrell D.J., Stauber G.B., Brown S., Cousens L.S., Stack R.J. (1993). Preparation of affinity-fractionated, heparin-derived oligosaccharides and their effects on selected biological activities mediated by basic fibroblast growth factor. J. Biol. Chem..

[59] Jastrebova N., Vanwildemeersch M., Rapraeger A.C., Gimenez-Gallego G., Lindahl U., Spillmann D. (2006). Heparan sulfate-related oligosaccharides in ternary complex formation with fibroblast growth factors 1 and 2 and their receptors. J. Biol. Chem..

[60] Plotnikov A.N., Schlessinger J., Hubbard S.R., Mohammadi M. (1999). Structural basis for FGF receptor dimerization and activation. Cell.

[61] Presta M., Dell’Era P., Mitola S., Moroni E., Ronca R., Rusnati M. (2005). Fibroblast growth factor/fibroblast growth factor receptor system in angiogenesis. Cytokine Growth Factor Rev..

[62] Zhang F., Zhang Z., Lin X., Beeken A., Eliseenkova A.V., Mohammadi M., Linhardt R.J. (2009). Compositional analysis on heparin/heparin sulfate interacting with FGF FGFR complexes. Biochemistry.

[63] Zhang F., Zheng L., Cheng S., Peng Y., Fu L., Zhang X., Lindardt R.J. (2019). Comparison of the interactions of different growth factors and glycosaminoglycans. Molecules.

[64] Marianayagam N.J., Sunde M., Mattews J.M. (2004). The power of two: Protein dimerization in biology. Trends Biochem. Sci..

[65] Goodger S.J., Robinson C.J., Murphy K.J., Gasiunas N., Harmer N.J., Blundell T.L., Pye D.A., Gallagher J.T. (2008). Evidence that heparin saccharides promote FGF2 mitogenesis through two distinct mechanisms. J. Biol. Chem..

[66] Atha D.H., Lormeau J.C., Petitoum M., Rosenberg R.D., Choay J. (1985). Contribution of monosaccharide residues in heparin binding to antithrombin III. Biochemistry.

[67] Atha D.H., Lormeau J.C., Petitou M., Rosenberg R.D., Choay J. (1987). Contribution of 3-*O*- and 6-*O*-sulfated glucosamine residues in the heparin-induced conformational change in antithrombin III. Biochemistry.

[68] Esko J.D., Lindahl U. (2001). Molecular diversity of heparan sulfate. J. Clin. Investig..

[69] Chavante S.F., Brito A.S., Lima M., Yates E., Nader H., Guerrini M., Torri G., Bisio A. (2014). A heparin-like glycosaminoglycan from shrimp containing high levels of 3-*O*-sulfated d-glucosamine groups in an unusual trisaccharide sequence. Carbohydr. Res..

[70] Kreuger A., Salmivirta M., Sturiale L., Gimenez-Gallego G., Lindahl U. (2001). Sequence analysis of heparan sulfate epitopes with graded affinities for fibroblast growth factors 1 and 2. J. Biol. Chem..

[71] Jemth P., Kreuger J., Kusche-Gullberg M., Sturiale L., Gimenez-Gallego G., Lindahl U. (2002). Biosynthetic oligosaccharide libraries for identification of protein-binding heparan sulfate motifs. Exploring the structural diversity by screening for fibroblast growth factor (FGF) 1 and FGF -2 binding. J. Biol. Chem..

[72] Schlessinger J., Plotnikov A.N., Ibrahimi O.A., Eliseenkova A.V., Yeh B.K., Yayon A., Linhardt R.J., Mohammadi M. (2000). Crystal structure of a ternary FGF-FGFR-heparin complex reveals a dual role for heparin in FGFR binding and dimerization. Mol. Cell.

[73] Allen B.L., Filla M.S., Rapraeger A.C. (2001). Role of heparin sulfate as a tissue-specific regulator of FGF-4 and FGF receptor recognition. J. Cell Biol..

[74] Feyzi E., Lustig F., Fager G., Spillmann D., Lindahl U., Salmivirta M. (1997). Characterization of heparin and heparan sulfate domains binding to the long splice variant of platelet-derived growth factor A chain. J. Biol. Chem..

[75] Abramsson A., Kurup S., Busse M., Yamada S., Lindblom P., Schallmeiner E., Stenzel D., Sauvaget D., Ledin J., Ringvall M. (2007). Defective *N*-sulfation of heparin sulfate proteoglycans limits PDGF-BB binding and pericyte recruitment in vascular development. Gene Dev..

[76] Sakata H., Stahl S.J., Taylor W.G., Rosenberg J.M., Sakaguchi K., Wingfield P.T., Rubin J.S. (1997). Heparin-binding and oligomerization of hepatocyte growth factor/scatter factor isoforms. J. Biol. Chem..

[77] Lyon M., Deakin J.A., Mizuno K., Nakamura T., Gallagher J.T. (1994). Interaction of hepatocyte growth factor with heparan sulfate. Elucidation of the major heparan sulfate determinants. J. Biol. Chem..

[78] Ashikari S., Habuchi H., Kimata K. (1995). Characterization of heparan sulfate oligosaccharides that bind to hepatocyte growth factor. J. Biol. Chem..

[79] Ono K., Hattori H., Takeshita S., Kurita A., Ishihara M. (1999). Structural features in heparin which interact with VEGF165 and modulate its biological activity. Glycobiology.

[80] Teran M., Nugent M.A. (2015). Synergistic binding of vascular endothelial growth factor-A and its receptors to heparin selectively modulates complex affinity. J. Biol. Chem..

[81] Liu J.R., Wang H.F., Yu D.F., Chen X.Y., He S.Y. (2017). Modulation of binding to vascular endothelial growth factor and receptor by heparin derived oligosaccharide. Carbohydr. Polym..

[82] Lyon M., Rushton G., Gallagher J.T. (1997). The interaction of the transforming growth factor-betas with heparin/heparan sulfate is isoform-specific. J. Biol. Chem..

[83] Rider C.C., Mulloy B. (2017). Heparin, heparin sulphate and the TGF-β superfamily. Molecules.

[84] McCaffrey T.A., Falcone D.J., Du B. (1992). Transforming growth factor-beta 1 is a heparin-binding protein: Identification of putative heparin-binding regions and isolation of heparins with varying affinity for TGF-beta 1. J. Cell. Physiol..

[85] Muramatsu T. (2010). Midkine, a heparin-binding cytokine with multiple roles in development, repair and diseases. Proc. Jpn. Acad. Ser. B.

[86] Zou P., Muramatsu H., Ichihara-Tanaka K., Habuchi O., Ohtake S., Ikematsu S., Sakuma S., Muramatsu T. (2003). Glucosaminoglycan structures reqired for strong binding to midkine, a heparin-binding growth factor. Glycobiology.

[87] Najjam S., Gibbs R.V., Gordon M.Y., Rider C.C. (1997). Characterization of human recombinant interleukin 2 binding to heparin and heparin sulfate using an ELISA approach. Cytokine.

[88] Mummery R.S., Rider C.C. (2000). Characterization of the heparin-binding properties of IL-6. J. Immunol..

[89] Nordsieck K., Baumann L., Hintze V., Pisabarro M.T., Schnabelrauch M., Beck-Sickinger A.G., Samsonov S.A. (2018). The effect of interleukin-8 truncations on its interactions with glycosaminoglycans. Biopolymers.

[90] Salek-Ardakani S., Arrand J.R., Shaw D., Mackett M. (2000). Heparin and heparin sulfate bind interleukin-10 and modulate its activity. Blood.

[91] Hasan M., Najjam S., Gordon M.Y., Gibbs R.V., Rider C.C. (1999). IL-12 is a heparin-binding cytokine. J. Immunol..

[92] Jayanthi S., Koppolu B.P., Nguyen K.G., Smith S.G., Felber B.K., Kumar T.K.S., Zaharoff D.A. (2017). Modulation of interleukin-12 activity in the presence of heparin. Sci. Rep..

[93] Maccarana M., Lindahl U. (1993). Mode of interaction between platelet factor 4 and heparin. Glycobiology.

[94] Stringer S.E., Gallagher J.T. (1997). Specific binding of the chemokine platelet factor 4 to the heparan sulfate. J. Biol. Chem..

[95] Sadir D., Forest E., Lortat-Jacob H. (1998). The heparin sulfate binding sequence of interferon-γ increased the rate of the interferon-γ-interferon-γ receptor complex formation. J. Biol. Chem..

[96] Sarrazin S., Bonnaffe D., Lubineau A., Lortat-Jacob H. (2005). Heparan sulfate mimicry: A synthetic glycoconjugate that recognizes the heparin binding domain of interferon-gamma inhibits the cytokine activity. J. Biol. Chem..

[97] Modrowski D., Lomri A., Marie P.J. (1998). Glycosaminoglycans bind granulocyte-macrophage colony-stimulating factor and modulate its mitogenic activity and signaling in human osteoblastic cells. J. Cell. Physiol..

[98] Sebollela A., Cagliari T.C., Limaverde G.S., Chapeaurouge A., Sorgine M.H., Ramos C.H., Ferreira S.T. (2005). Heparin-binding sites in granulocyte-macrophage colony-stimulating factor. Localization and regulation by histidine ionization. J. Biol. Chem..

[99] Higashiyama S., Lau R., Bener G.E., Abraham J.A., Klagsbrun M. (1992). Structure of heparin-binding EGF-like growth factor. J. Biol. Chem..

[100] Douglus M.S., Ali S., Rix D.A., Zhang J.-G., Kirby J.A. (1997). Endothelial production of MCP-1: Modulation by heparin and consequences for mononuclear cell activation. Immunology.

[101] Lau E.K., Paavola C.D., Johnson Z., Gaudry J.-P., Geretti E., Borlat F., Kung A.J., Proudfoot A.E., Handel T.M. (2004). Identification of glycosaminoglycan binding site of the CC chemokine, MCP-1. J. Biol. Chem..

[102] Kishimoto S., Nakamura S., Hattori H., Nakamura S.-I., Oonuma F., Kanatani Y., Tanaka Y., Mori Y., Harada Y., Tagawa M. (2009). Human stem cell factor (SCF) is a heparin-binding cytokine. J. Biochem..

[103] Sally E., Nelson M.S., Gupta P. (2003). Identification of an MIP-1α-binding heparin sulfate oligosaccharide that support long-term in vitro maintenance of human LTC-ICs. Blood.

[104] Koopmann W., Ediriwickrema C., Krangel M.S. (1999). Structure and function of the glycosaminoglycan binding site of chemokine macrophage-inflammatory protein-1β. J. Immunol..

[105] Maccarana M., Casu B., Lindahl U. (1993). Minimal sequence in heparin/heparan sulfate required for binding of basic fibroblast growth factor. J. Biol. Chem..

[106] Turnbull J.E., Fernig D.G., Ke Y., Wilkinson M.C., Gallagher J.T. (1992). Identification of the basic fibroblast growth factor binding sequence in fibroblast heparan sulfate. J. Biol. Chem..

[107] Yu H., Munoz E.M., Edens R.E., Linhardt R.J. (2005). Kinetic studies on thwe interactions of heparin and complement proteins using surface plasmon resonance. Biochim. Biophys. Acta.

[108] Hsiao F.S., Yang S.K., Lin J.M., Chen Y.W., Chen C.S. (2019). Protein interacome analysis of iduronic acide-containing glycosaminoglycans reveals a novel flagellar invasion factor MbhA. J. Proteomics..

[109] Casu B., Petitou M., Provasoli M., Sinay P. (1998). Conformational flexibility: A new concept for explaining binding and biological properties of iduronic acid-containing glycosaminoglycans. Trends Biochem. Sci..

[110] Gunay N.S., Linhardt R.J. (1999). Heparinoids: Structure, biological activities and therapeutic applications. Planta Med..

[111] Casu B., Naggi A., Torri G. (2010). Heparin-derived heparin sulfate mimics to modulate heparin sulfate-protein interaction in inflammation and cancer. Matrix Biol..

[112] Pellegrini L., Burke D.F., von Delft F., Mulloy B., Blundell T.L. (2000). Crystal structure of fibroblast growth factor receptor ectodomain bound to ligand and heparin. Nature.

[113] Ruoslahi E., Yamaguchi Y. (1991). Proteoglycans as modulators of growth factor activities. Cell.

[114] Nader H.B., Dietrich C.P., Buonassisi V., Colburn P. (1987). Heparin sequences in the heparin sulfate chains of an endothelial cell proteoglycan. Proc. Natl. Acad. Sci. USA.

[115] Park P.W., Reizes O., Bernfield M. (2000). Cell surface heparin sulfate proteoglycans: Selective regulators of ligand-receptor encounters. J. Biol. Chem..

[116] Gallagher J. (2015). Fell-Muir Lecture: Haparan sulphate and the art of cell regulator: A polymer chain conducts the protein orchestra. Int. J. Exp. Pathol..

[117] Hirsh J., Warkentin T.E., Shaughnessy S.G., Anand S.S., Halperin J.L., Raschke R. (2001). Heparin and low-molecular heparin, mechanisms of action, pharmacokinetics, dosing, monitoring, efficacy, and safety. Chest.

[118] Fransson L.A. (1978). Periodate oxidation of d-glucuronic acid residues in heparan sulfate and heparin. Carbohydr. Res..

[119] Fransson L.A., Carlstedt I. (1974). Alkaline and Smith degradation of oxidized dermatan sulfate-chondroitin sulfate copolymers. Carbohydr. Res..

[120] Lin C.C., Metters A.T. (2006). Hydrogels in controlled release formulations: Network design and mathematical modeling. Adv. Drug Deliv. Rev..

[121] Wang S.C., Chen B.H., Wang L.F., Chen J.S. (2007). Characterization of chondroitin sulfate and its interpenetrating polymer network hydrogels for sustaining-drug release. Int. J. Pharmcol..

[122] Chu H.H., Gao J., Chen C.W., Huard J., Wang Y.D. (2011). Injectable fibroblast growth factor-2 coacervate for persistent angiogenesis. Proc. Natl. Acad. Sci. USA.

[123] Fujita M., Ishihara M., Shimizu M., Obara K., Ishizuka T., Saito Y., Yura H., Morimoto Y., Takase B., Matsui T. (2004). Vascularization in vivo caused by the controlled release of fibroblast growth factor-2 from an injectable chitosan/non-anticoagulant heparin hydrogel. Biomaterials.

[124] Fujita M., Ishihara M., Shimizu M., Obara K., Nakamura S., Kanatani Y., Morimoto Y., Takase B., Matui T., Kikuchi M. (2007). Therapeutic angiogenesis induced by controlled release of fibroblast growth factor-2 from injectable chitosan/non-anticoagulant heparin hydrogel in rat hind limb ischemia model. Wound Repair Regen..

[125] Nakamura S., Ishihara M., Obara K., Masuoka K., Ishizuka T., Kanatani Y., Takase B., Matsui T., Hattori H., Sato T. (2006). Controlled release of fibroblast growth factor-2 from injectable 6-*O*-desulfated heparin hydrogel and subsequent effect on in vivo vascularization. J. Biomed. Mater. Res. A.

[126] Nakamura S., Nambu M., Ishizuka T., Hattori H., Kanatani Y., Kishimoto S., Amano Y., Aoki H., Kiyosawa T., Ishihara M. (2008). Effect of fibroblast growth factor-2 from chitosan/fucoidan micro complex hydrogel on in vitro and in vivo neovascularization. J. Biomed. Mater. Res. A.

[127] Rele S.M., Cui W., Wang L., Hou S., Barr-Zarse G., Tatton D., Gnanou Y., Esko J.D., Chaikof E.L. (2005). Dendrimer-like PEO glycopolymer exihibit anti-infmammatory properties. J. Am. Chem. Soc..

[128] Paluck S.J., Nguyen T.H., Maynard H.D. (2016). heparin-mimicking polymers: Synthesis and biological applications. Biomacromolecules.

[129] Ishihara M., Saito Y., Yura H., Ono K., Ishikawa K., Hattori H., Akaike T., Kurita A. (2000). Heparin-carrying polystyrene to mediate cellular attachment and growth via interaction with growth factors. J. Biomed. Mater. Res. A.

[130] Ishihara M., Ono K., Ishikawa K., Hattori H., Saito Y., Yura H., Akaike T., Ozeki Y., Tanaka S., Mochizuki H. (2000). Enhanced ability of heparin-carrying polystyrene (HCPS) to inhibit growth factor-induced endothelial cell growth. J. Biochem..

[131] Ono K., Ishihara M., Ishikawa K., Ozeki Y., Deguchi H., Sato M., Hashimoto H., Saitoh Y., Yura H., Kurita A. (2002). Periodate-treated, non-anticoagulant heparin carrying polystyrene (NAC-HCPS) affects angiogenesis and inhibits subcutaneous induced tumor growth and metastasis to the lung. Br. J. Cancer.

[132] Fujita M., Ishihara M., Ono K., Matsumura K., Saito Y., Yura H., Morimoto Y., Shimizu M., Takase B., Ozeki S. (2004). Inhibition of neointimal proliferation in balloon-injured arteries using by non-anticoagulant heparin-carrying polystyrene (NAC-HCPS). J. Cardiovasc. Pharmacol..

[133] Kobayashi A., Sumitomo H., Ina A. (1985). Synthesis and functions of polystylene derivatives having pendant oligosaccharides. Polym. J..

[134] Hattori H., Nogami Y., Tanaka T., Amano Y., Fukuda K., Kishimoto S., Kanatani Y., Nakamura S., Takase B., Ishihara M. (2008). Expansion and characterization of adipose tissue-derived stromal cells cultured with low serum medium. J. Biomed. Mater. Res. B.

[135] Ishihara M., Sato M., Hattori H., Saito Y., Yura H., Ono K., Masuoka K., Kikuchi M., Fujikawa K., Kurita A. (2001). Heparin-carrying polystyrene (HCPS)-bound collegen substratum to immobilize heparin-binding growth factors and to enhance cellular growth. J. Biomed. Mater. Res..

[136] Kulkarni A.D., Vanjari Y.H., Sancheti K.H., Patel H.M., Belgamwar V.S., Surana S.J., Pardeshi C.V. (2016). Polyelectrolyte complexes: Mechanisms, critical experimental aspects, and applications. Artif. Cells Nanomed. Biotechnol..

[137] Berger J., Reist M., Mayer J.M., Felt O., Peppas N.A., Gurny R. (2004). Structure and interactions in covalently and ionically crosslinked chitosan hydrogels for biomedical applications. Eur. J. Pharm. Biopharm..

[138] Ishihara M., Kishimoto S., Nakamura S., Sato Y., Hattori H. (2019). Polyelectrocyte complexes of natural polymers and their biomedical applications. Polymers.

[139] Khurshid H., Kim S.H., Bonder M.J., Colak L., Ali B., Shah S.I., Kiick K.L., Hadjipanayis G.C. (2009). Development of heparin-coated magnetic nanoparticles for targeted drug delivery applications. J. Appl. Phys..

[140] Huang H., Yang X. (2004). Synthesis of polysaccharide-stabilized gold and silver nanoparticles: A green method. Carbohydr. Res..

[141] Chauvierre C., Marden M.C., Vauthier C., Labarre D., Couvreur P., Leclerc L. (2004). Heparin-coated poly (alkylcyanoacrylate) nanoparticles coupled to hemoglobin: A new oxygen carrier. Biomaterials.

[142] Park K., Lee G.Y., Kim Y.S., Yu M., Park R.W., Kim I.S., Kim S.Y., Byun Y. (2006). Heparin-deoxycholic acid chemical conjugate as an anticancer drug carrier and its antitumor activity. J. Control. Release.

[143] Jang E., Albadawi H., Watkins M.T., Edelman E.R., Baker A.B. (2012). Syndecan-4 proteoliposomes enhance fibroblast growth factor-2 (FGF-2)-induced proliferation, migration, and neovascularization of ischemic muscle. Proc. Natl. Acad. Sci. USA.

[144] Hagiwara K., Kishimoto S., Ishihara M., Koyama Y., Mazda O., Sato T. (2013). In vivo gene transfer using pDNA/chitosan/chondroitin sulfate ternary complexes: Influence of chondroitin sulfate on the stability of freeze-dried complexes and transfer gene expression in vivo. J. Gene Med..

[145] Houska M., Brynda E., Bahata K. (2004). The effect of polyelectrolyte chain length on layer-by layer protein/polyelectrolyte assembly—An experimental study. J. Colloid Interface Sci..

[146] Seyrek E., Dubin P. (2010). Glycosaminoglycans as polyelectrolytes. Adv. Colloid Interface.

[147] Wolzt M., Wetermann A., Nieszpaur-Los M., Schneider B., Fassolt A., Lechner K., Kyrle P.A. (1995). Studies on the neutralizing effects of protamine on unfractionated and low molecular weight heparin (Fragmin^®^) at the site of activation of the coagulation system in man. Thromb. Haemost..

[148] Pan M., Lezo J.S., Medina A., Romero M., Hernandez E., Segura J., Melian F., Wanguemert F., Landin M., Benitez F. (1997). In-laboratory removal of femoral sheath following protamine administration in patients having intracoronary stent implantation. Am. J. Cardiol..

[149] Mori Y., Nakamura S., Kishimoto S., Kawakami M., Suzuki S., Matsui T., Ishihara M. (2010). Preparation and characterization of low-molecular-weight heparin/protamine nanoparticles (LMW-H/P NPs) as FGF-2 carrier. Int. J. Nanomed..

[150] Nakamura S., Kanatani Y., Kishimoto S., Nambu M., Ohno C., Hattori H., Fujita M., Hattori H., Tanaka Y., Kiyosawa T. (2009). Controlled release of FGF-2 using fragmin/protamine microparticles and effect on neovascularization. J. Biomed. Mater. Res. A.

[151] Kishimoto S., Ishihara M., Nakamura S., Takikawa M., Fujita M., Sumi Y., Kiyosawa T., Sato T., Kanatani Y. (2013). Fragmin/protamine microparticles to absorb and protect HGF and to function as local HGF carrier in vivo. Acta Biomaterilia.

[152] Nemeno J.G.E., Lee S., Yang W., Lee K.M., Lee J.I.K. (2014). Applications and implications of heparin and protamine in tissue engineering and regenerative medicine. Biomed. Res. Int..

[153] Takikawa M., Nakamura S.-I., Nakamura S., Nambu M., Ishihara M., Fujita M., Kishimoto S., Doumoto T., Yanagibayashi S., Azuma R. (2011). Enhancement of vascularization and granulation tissue formation by growth factors in human platelet-rich plasma-containing fragmin/protamine microparticles. J. Biomed. Mater. Res. B.

[154] Horio T., Fujita M., Tanaka Y., Ishihara M., Kishimoto S., Nakamura S., Hase K., Maehara T. (2011). Efficacy of fragmin/protamine microparticles containing fibroblast growth factor-2 (F/P MPsFGF-2) to induce collateral vessels in a rabbit model of hindlimb ischemia. J. Vasc. Surg..

[155] Nakamura S., Ishihara M., Takikawa M., Kishimoto S., Isoda S., Fujita M., Sato M., Maehara T. (2012). Attenuation of limb loss in an experimentally induced hindlimb ischemic model by fibroblast growth factor-2/fragmin/protamine microparticles as a delivery system. Tissue Eng. Part A.

[156] Fujita M., Horio T., Kishimoto S., Nakamura S., Takikawa M., Nakayama T., Yamamoto Y., Shimizu M., Hattori H., Tachibana S. (2012). Effects of platelet-rich plasma-containing fragmin/protamine microparticles in enhancing endothelial and smooth muscle cell growth and inducing collateral vessels in a rabbit model of hindlimb ischemia. J. Biomed. Mater. Res. B..

[157] Takabayashi Y., Mambu M., Ishihara M., Kuwabara M., Fukuda K., Nakamura S., Hattori H., Kiyosawa T. (2016). Enhanced effect of fibroblast growth factor-2-containing dalteparin/protamine nanoparticles on hair growth. Clin. Cosmet. Investig. Dermatol..

[158] Takikawa M., Nakamura S.-I., Nakamura S., Nambu M., Ishihara M., Murakami K., Kishimoto S., Sasaki K., Yanagishita S., Azuma R. (2011). Enhanced effect of platelet-rich plasma containing a new carrier on hair growth. Dermatol. Surg..

[159] Takikawa M., Ishihara M., Takabayashi Y., Sumi Y., Takikawa M., Yoshida R., Nakamura S., Hattori H., Yanagibayashi S., Yamamoto N. (2015). Enhanced healing of mitomycin C-treated healing-impaired wounds in rats with PRP-containing fragmin/protamine microparticles (PRP&F/P MPs). J. Plast. Surg. Hand Surg..

[160] Kinoda J., Ishihara M., Nakamura S., Fujita M., Fukuda K., Sato Y., Yokoe H. (2018). Protective effect of FGF-2 and low-molecular-weight heparin/protamine nanoparticles on radiation-induced healing-impaired wound repair in rats. J. Radiat. Res..

[161] Takikawa M., Ishihara M., Kishimoto S., Nakamura S., Yanagibayashi S., Hattori H., Azuma R., Yamamoto N., Kiyosawa T. (2011). PRP&F/P MPs improved survival of dorsal paired pedicle skin flaps in rats. J. Surg. Res..

[162] Takabayashi Y., Ishihara M., Sumi Y., Takikawa M., Nakamura S., Kiyosawa T. (2015). Platelet-rich plasma-containing fragmin-protamine micro-nanoparticles promote epithelialization and angiogenesis in split-thickness skin graft donor sites. J. Surg. Res..

[163] Takikawa M., Nakamura S., Ishihara M., Takabayashi Y., Fujita M., Hattori H., Kushibiki T., Ishihara M. (2015). Improved angiogenesis and healing in crush syndrome by fibroblast growth factor-2-containing low-molecular-weight heparin (Fragmin)/protamine nanoparticles. J. Surg. Res..

[164] Wu P.-I.K., Edelman E.R. (2008). Structural biomechanics modulate intramuscular distribution of locally delivery drug. J. Biomech..

[165] Kumano I., Kishimoto S., Nakamura S., Hattori H., Tanaka Y., Nakata M., Sato T., Fujita M., Maehara T., Ishihara M. (2011). Fragmin/protamine microparticles (F/P MPs) as cell carriers enhance the formation and growth of tumors in vivo. Cell. Mol. Bioeng..

[166] Volpe J.P., Milasm L. (1990). Influence of tumor transplantation methods on tumor growth rate and metastatic potential of solitary tumors derived metastasis. Clin. Exp. Metasitasis.

[167] Nakamura S., Kishimoto S., Nakamura S.I., Nambu M., Fujita M., Tanaka Y., Mori Y., Tagawa M., Maehara T., Ishihara M. (2010). Fragmin/protamine microparticles as cell carriers to enhance viability of adipose-derived stromal cells and their subsequent effect on in vivo neovascularization. J. Biomed. Mater. Res. A.

[168] Kishimoto S., Ishihara M., Mori Y., Takikawa M., Hattori H., Nakamura S., Sato T. (2013). Effective expansion of human adipose-derived stromal cells and bone marrow-derived mesenchymal stem cells cultured on a fragmin/protamine nanoparticles-coated substratum with human platelet-rich plasma. J. Tissue Eng. Regen. Med..

[169] Kishimoto S., Nakamura S., Nakamura S.I., Kanatani Y., Hattori H., Tanaka Y., Harada M., Tagawa M., Mori Y., Maehara T. (2009). Fragmin/protamine microparticle-coated matrix immobilized cytokines to stimulate various cell proliferations with low serum media. Artif. Organs.

[170] Kishimoto S., Nakamura S., Nakamura S.I., Hattori H., Oomuma F., Kanatani Y., Tanaka Y., Harada Y., Tagawa M., Maehara T. (2009). Cytokine-immobilized microparticle-coated plates for culturing hematopoietic progenitor cells. J. Control. Release.

[171] Kishimoto S., Ishihara M., Takikawa M., Takikawa M., Sumi Y., Nakamura S., Fujita M., Sato T., Kiyosawa T. (2014). Three-dimensional culture using human plasma-medium gel with fragmin/protamine microparticles for proliferation of various human cells. Cytotechnology.

[172] Kishimoto S., Ishihara M., Mori Y., Takikawa M., Sumi Y., Nakamura S., Sato T., Kiyosawa T. (2012). Three-dimensional expansion using plasma-medium gel with fragmin/protamine nanoparticles and FGF-2 to stimulate adipose-derived stromal cells and bone marrow-derived mesenchymal stem cells. BioRes. Open Access.

[173] Rokstad A.M., Donati I., Borgogna M., Oberholzer J., Strand B.L., Espevikm T., Skjak-Braek G. (2006). Cell-compatible covalently reinforced beads obtained from a chemoenzymatically engineered alginate. Biomaterials.

[174] Wei Z., Zhao J., Chen Y.M., Zhang P., Zhang Q. (2016). Self-healing polysaccharide-based hydrogels as injectable carriers for neural stem cells. Sci. Rep..

[175] Sumi Y., Ishihara M., Kishimoto S., Takikawa M., Doumoto T., Azuma R., Nakamura S., Hattori H., Fujita M., Kiyosawa T. (2013). Transplantation of inbred adipose-derived stromal cells in rats with plasma gel containing fragmin/protamine microparticles and FGF-2. J. Biomed. Mater. Res. B.

[176] Sumi Y., Ishihara M., Kishimoto S., Takikawa M., Hattori H., Takikawa M., Doumoto T., Azuma R., Nakamura S., Fujita M. (2014). Effective wound healing in streptozotocin-induced diabetic rats by adipose-derived stromal cell-transplantation in plasma-gel containing fragmin/protamine microparticles. Ann. Plast. Surg..

[177] Baker A.B., Gibson W.J., Kolachalama V.B., Golomb M., Indolfi L., Spruell C., Zcharia E., Vlodavsky I., Edelman E.R. (2012). Heparanase regulates thrombosis in vascular injury and stent induced flow disturbance. J. Am. Coll. Cardiol..

[178] Biran R., Pond D. (2017). Heparin coatings for improving blood compatibility of medical devices. Adv. Drug Deliv. Rev..

[179] Wendel H.P., Ziemer G. (1999). Coating-techniques to improve the hemocompatibility of artificial devices used for extracorponeal circulation. Eur. J. Cardio Thorac. Surg..

[180] Tanzi M.C. (2005). Bioactive technologies for hemocompatibility. Expert Rev. Med. Devices.

[181] Murugesan S., Xie J., Linhardt R.J. (2008). Immobilization of heparin: Approaches and applications. Curr. Top. Med. Chem..

[182] Hwang C.W., Wu D., Edelman E.R. (2001). Physiological transport forces govern drug distribution for stent-based delivery. Circulation.

[183] Obara K., Ishihara M., Ozeki Y., Ishizuka T., Hayashi T., Nakamura S., Saito Y., Yura H., Matsui T., Hattori H. (2005). Controlled release of paclitaxel from photocrosslinked chitosan hydrogels and its subsequent effect on subcutaneous tumar growth in mice. J. Control. Release.

[184] Gore S., Andersson J., Biran R., Underwood C., Riesenfeld J. (2014). Heparin surfaces: Impact of immobilization chemistry on hemocompatibility and protein adsorption. J. Biomed. Mater. Res. B.

[185] Islam T., Butler M., Sikkander S.A., Toida T., Linhardt R.J. (2002). Further evidence that periodate cleavage of heparin occurs primarily through the antithrombin binding site. Carbohydr. Res..

